# Report on the History of Medicine

**Published:** 1867-04

**Authors:** S. J. Young

**Affiliations:** Ch’n Com., Paris, Ill.


### CHICAGO MEDICAL EXAMINER.

N. S. DAVIS, M.D., Editor.

VOL. VIII. APRIL, 1867. NO. 4.

### Original Contributions.

### ARTICLE XVII.

### REPORT ON THE HISTORY OF MEDICINE.

By S. J. YOUNG, M.D., Ch'n Com., Paris, Ill.

Read to the Esculapian Society, at the Meeting held at Paris, Ill., May, 1866.

The practice of medicine implies the application of science to the cure of diseases and to their prevention; while that of surgery, one of its branches, relates to a similar application to the accidents of the human body and all that class of diseases requiring the use of the knife, or those more purely objective—fractures, dislocations, ulcers, etc. This is our understanding at the present time, and has been since the increase of knowledge entitled it to a science. But, in the early ages of the human family, it could not claim this rank; and whatever medicinal agents were then used were merely from experience, as not even the virtues of the simplest herb or plant could have been known in any other way.

It is evident that in all ages agents of one kind or another have been used for the relief of those afflicted from bodily disease or injury, and that some would give more attention to it than others, and would gradually become the doctors or prescribers of medicine; and, as the human mind loves mystery, they made up in their agents what they lacked in knowledge—and the doctor and all he knew was a bundle of unfathomable mystery. In the mysterious department we have not changed, as this class is as much sought after now as ever before, and spiritual and magical doctors have a lengthened train of followers, who believe everything they cannot see, and nothing which they can.

They avoid the *quod erat demonstrandum*, and hug to their foolish bosoms the *non est demonstrandum.*

But to this small beginning, one fact has been added to another, one demonstration to another, until a vast structure has been raised up, now almost perfect, to illuminate the world and gladden the human heart. From the depths of the ocean, a tiny insect has built up in increasing labor vast reefs of coral, each brief age having its increase, giving monuments exceeding those of man. The flowing and the ebbing billows of the sea have thrown up, sand by sand, islands which have become the abode of man. Three thousand years ago, and more, the shapeless mass of medicine began to assume form. Ages anterior to that, in Egypt and China, it was practiced as an art; and when the inhabitants of Greece were but savages, living in caves and upon acorns, Phœnicia boasted of her Tyre and the arts of civilization, and the banks of the Nile gave to Athens her foundation, and name, and the arts and sciences, and Thebes sprang up from her no less civilized neighbors, the Phœnicians. From revolutions, came this new world, so long the seat of empire in art and science, and all that was great and commanding.

Æsculapius, whether mythical or real, was the representative of the art of medicine, became a god, to whom temples were erected and in which he was worshipped. The serpent and the cock were second to him—and these figures are still emblems of our art. He lived before the Trojan war; was said to have been the son of Apollo; accompanied the Argonautic expedition, as their physician; and was greatly skilled in the power of medical plants. His knowledge led to his death, for Plato complained that he restored so many to life, that this god (of the infernal regions) had nothing to do, and Æsculapius was killed by Jupiter by a thunderbolt, the forger of which, Cyclops, was killed by the incensed father, Apollo. This gives the highest evidence of his skill, and is the only instance known where the grave has made this complaint. Unlike our great father, we shall have the credit of giving to the grave all that is due.

Human skill has bounds, and life a termination, but to us belongs the duty of giving length to that which might be short, and ease to that which might be painful; and thus far our profession excels all others. It is as much superior to that of law, as human life is of more value than property. Voltaire says, that a good physician is far above the grand of the world, and kindred to divinity.

Machaon and Podalirius, sons of Æsculapius, accompanied the Greeks to the Trojan war, as physicians. The former was a skilful surgeon, and devoted his time to the dressing of wounds. The latter was invited to stay a pestilence in the Grecian camp, which had baffled the skill of others. On his return from the war, being shipwrecked, he cured the king's daughter, who had fallen from the house and was insensible, by bleeding her. This is the first recorded case of bleeding being applied to one stunned by a fall. Having cured her, he married her. Philoctetes was cured of a wound of the foot, by Machaon, and in a manner which would not be discreditable to the surgery of this age. He was placed in a bath and cast into a deep sleep, when the putrified flesh was cut out, was washed with wine, and herbs applied, by which the parts were healed and the hero restored to health. The statement of this fact is evidence of respectable surgical knowledge—precisely what would now be done, in a similar case, by every gentleman of this Society.

Pindar states, that Æsculapius cured ulcers, wounds, fevers, etc., by enchantments, calming potions, incisions, and by external applications. This was pretty good eclectic practice, and rather better than that adopted by the mendicant tribe of the present day. It certainly compared favorably with the charms used by the people of Devonshire, England, 150 years ago. Their infallible remedy for sciatica, called "bone shave,"

was this:—The patient must lie on his back, on the bank of a river or brook, with a straight staff by his side, and must have the following words repeated over him:—

"Bone shave right,
"Bone shave straight.
"As the water runs by the stave,
"Good for bone shave."

In the name of the patient repeated.

The interval between the two methods is nearly 2000 years, and the former, I think, has the preference. Instinct and experience go hand in hand in the beginning of the healing art. Instinct would lead the feverish to quench thirst with cooling drinks; one with ague, to get warm by fire and covering; to reject food when the stomach would not receive it. And experience would reäpply those remedies which had been found efficacious. These fragments were gathered up in the primitive age, and went far towards the development of system.

After the death of Æsculapius, and he had become deified and temples had been erected to his memory, the medical art passed into the hands of the priests of these temples, who were called Æsculapiades. They purged, and bled, and vomited, and paid great attention to hygiene. Everything was involved in mystery, and nothing pertaining to the plans was revealed. It was a sacred and secret society, but knowledge was taught to those who desired to become priests. This age continued for nearly 700 years, when the secrecy was broken through and a new and enlightened age came, which was denominated the Philosophic Age. The Æsculapiades still continued to practice and teach medicine in the temples, but the pupils of Pythoras and of the Gymnasia went out and practiced, going from house to house, and city to city. The veil of secrecy was removed, and in the philosophic age a more enlightened and substantial foundation was laid. There were public discussions and teachings. This age continued about 200 years, and until the establishment of the Alexandrian library, 320 years before Christ. In this age lived Hippocrates, who wrote extensively upon everything pertaining to medicine, then known. His writings are frequently referred to at the present day, and exhibit the character of a student and philosopher, and a great advancement in medicine over the preceding ages. His doctrines and teachings were founded upon interpretation of the most abstruse subjects, and no facts or doctrines were announced without a reason, no matter how absurd. He had some knowledge of anatomy, physiology, and pathology, and has exercised a greater influence in medicine than any man that has ever lived since or had before. His school at Cos, acquired great reputation, and although the prejudice of the age in which he lived, forbade dissection of the human body, he obtained, from the dissection of animals, a general idea of anatomy, which enabled him to elucidate many of the doctrines he advocated. His doctrine of coction and vital forces, and of critical days or crisis in disease founded upon that, has undergone but few changes, and has received the sanction of the profession to a certain extent, since his time, now more than two thousand years. Whether false or true, it is a widely obtained belief that the vital forces are expended, or become dominant, and lead to life or death on certain days—on the 7th, 14th, 21st, or other days. It is a doctrine which has been taught all of us. It is a doctrine of truth and of nature, for whatever has life, terminates in death, and if three-score years and ten expends the vital forces unaided by disease, and lead to death, the presence of disease may expend them in a few hours, or days, or when the contest between the two are more equally balanced, it seems reasonable that a certain or fixed time will give dominance to one or the other. All experience teaches this. The eruptive diseases are clearly obedient to this law; and while I do not give credence to all that is said of critical days, I yield a belief to the principle, for it is a seal of God impressed upon all living matter. Some now before me have been born since one of the requirements in the medical schools of France was to understand the aphorisms of Hippocrates, and no man can understand them without being enlightened in his profession. To him and his family the science of medicine owes much of its success and usefulness.

His published works astonished the world, and made the physical science of man among the most important branches of philosophy. He was great and renowned in his own age, and the habit of calling medicine the art of Æsculapius was gradually lost, and the learned more frequently spoke of it as the science of Hippocrates. So popular did he make the study of medicine that no man was esteemed learned without it; and Plato and Aristotle were not only philosophers, but were eminent physicians and naturalists. The latter founded the first known museum of natural history upon earth. He was the protege of Philip, of Macedonia, the most enlightened and munificent of Kings, and when he selected the philosopher and physician as preceptor to his son, Alexander, he sent him, from his camps in Asia, the rarest of animals and plants to enrich the museum. How great this age of heroes, of philosophers, of statesmen, and of physicians! It was an age of thought, of reason, and of contest of mind, and rises sublimely beautiful above the grosser eras of succeeding ages. From the pupil and philosopher, Alexander rapidly became the king and conquerer, and in all his rapid strides to monarch of the earth, never forgot what was due to the advancement of science, and shortly after his death, a new era dawned upon the world, which to the ancients had a similar influence to the discovery of printing to the moderns. It was the formation of two great libraries—that of Pergamos and Alexandria—the former containing two hundred thousand volumes, and the latter six hundred thousand. These were accessible to all who sought their accumulated richness, and has conferred immortality on their founders. Their manuscripts were upon Egyptian papyrus, but a rivalry between them interdicted the exportation of the papyrus from Egypt to Pergamos, and the latter invented the parchment which has since been so extensively used, and upon which our diplomas of the present day are written. Technical or classical name of which is *charta pergamia.* When the beautiful Cleopatria had conquered Pergamos, she transferred its library to Alexandria, which for a thousand years continued the seat of learning. This great metropolis of medical science, and its medical schools, were to the ancients what the Paris schools are to the moderns. It was here that the Hebrew savans were charged by Philadelphus with the translation of the Bible into Greek, which has been so highly esteemed ever since, and is called the Septuagent. It was from the banks of the Nile that science and learning went to Greece, and the fire of genius died out at its fountain, when its brilliant light illuminated Attica and the world. After long ages its smouldering spark was again kindled in Egypt, which gave to Alexandria a name and rank which long survived the destruction of the kingdom in which it was located. It was here that anatomy was first taught by dissection. It was the great school of medicine for the world. It was here that Galen, a native of Pergamos, and the great physician of Rome studied. Criminals, who were executed, were given for dissection, and prejudice yielded to reason. The Ptolemies were its patrons, and its name was respected and honored in the uttermost ends of the earth. For four hundred years anatomy was taught in this school, and it may be inferred that it gave an immense impetus to medicine. In fact, the practice of that day was about as it was thirty years ago in this country. Bleeding, purging, and sweating were boldly used, and diseases were classified into internal, external, acute, and chronic. The treatment was divided into hygienie, pharmaceutic, and surgical. They gave great attention to symptons, and were remarkably exact in prognosis. Various sects arose at different periods of the Alexandrian school, which gave rise to discussions and the general improvement of medicine; but all, in reality, were based upon the Hippocratic doctrine, which consisted in employing such curative means as would be contrary to the generative cause of disease, or to the primitive and essential, to which all the other symptons are connected. When Alexandria fell under the Roman dominion, its schools of medicine lost their character; for anatomy could no longer withstand the Roman respect and prejudice for the dead, and about the second century of the Christian era its dissecting rooms were closed.

Hospitals were established about the fourth century, and the first in that holy city where our Savior was crucified, and by a Christian female from Rome. From that to this day, their holy blessings have been felt throughout the Christian world, and the maimed, and the halt, and the blind, have found within their sacred walls a holy rest of peace, which but for Christianity, which but for medicine, and for the charity of woman's heart, would have been forever shut out. But, in the destruction of the Alexandrian library, in the sixth century, passed away the glory of accumulated ages. For eighteen hundred years it was the great commercial city of the earth. But only for a thousand years from its foundation to its capture by the Saracens was it great in letters. With that event passed away its schools of philosophy, medicine, theology, and astronomy. The glory and empire of Greece and Rome had passed into the hands of the infidel, who, in their turn, contributed largely to the cultivation and spread of science and letters, and from the tenth to the thirteenth centuries, during Moorish rule in Spain, its schools at Cordova, Toledo, and Seville, were the resort of all Europe for instruction. They especially cultivated medicine, and had a school of celebrity at Bagdad, where Rhazes gave lectures, and whose name we *see* frequently quoted in modern works, especially in eruptive diseases. We can readily judge how justly great he is reputed, from a few sentences he has left us on the selection of a physician, and which is worthy of being understood by all: "Study carefully the antecedents of the man to whose care you propose confiding all you hold most dear upon earth; that is to say, your health, your life, and the health and lives of your wife and children. If the man is dissipating his time in frivolous pleasures; if he cultivates, with too much zeal, the arts that are foreign to his profession, such as music and poetry; still more, if he is addicted to wine and debauchery, refrain from committing into such hands a trust so precious. He merits your confidence who, having early applied himself to the study of medicine, has sought skilful instructors, and seen much disease; who has united to the assiduous reading of good authors his personal observations, for it is impossible to see everything and try everything in one's own practice, and the knowledge and experience of a single individual compared to the knowledge and skill of all men of all ages, resembles a slender brook of water that flows by the side of a great river." The Arabian physicians made improvements in pathology and therapeutics. They were the first to distinguish eruptive fevers by the external character of the eruption, variola and varicella. They introduced mild purgatives, such as cassia senna and manna, which happily took the place of the more drastic of the ancients. They also gave us the confection of syrups, of distilled waters, of tinctures, and compounded many new ointments, pomades, and plasters, for external applications.

A little further on, and during the middle ages, when the northern hordes had swept away the civilization of the world, and had destroyed empires, kingdoms, and cities, medicine again fell into the hands of the priesthood, and continued until it was so abused that the Pope interdicted the practice of the art in about the thirteenth century. For four centuries prior to that, the healing art was monopolized by the Jews and priests, and but little advancement was made, as may be inferred from the receipt of a priest, sold for the preparation of green frogs for medicinal use. It was a secret compound, and the vendor boasted of the large price he obtained for it. The priests and Jews stood at the head of the profession, but others of a lower order divided the profits with them. Such creatures as barbers, bath-tenders, and old women; especially did the barbers take under their charge minor operative surgery. The result of this condition in medicine so lowered it in the estimation of those having authority, that they began to make exactions of knowledge upon those who practiced the profession. Medical colleges again came into repute, and were founded at various points throughout Europe. That of Salerno, in Sicily, obtained great reputation. But its epoch of greatest splendor was about the tenth century, when the crusaders of Christian Europe were rushing to the Holy Land. Being located on this great pathway of travel, gave it advantages of giving aid to the sick and wounded which others less favorably situated did not possess, and its healthy and delicious climate made it a desirable resort for kings and knights, and barons, in their crusades. In fact, its reputation drew from all parts of Europe those afflicted with diseases and wounds difficult of cure. The son of William the Conqueror, was treated there for a wound on his return from the crusade. Its faculty issued edicts for the preservation of health, which were sought after and respected by crowned heads. To the king of England they wrote: "If you wish good health, banish despondency and avoid anger. Drink but little wine, eat light suppers, and do not disdain to take some exercise after meals. Do not sleep during the day; (do not retain too long the urine and evacuations from the bowels.) By observing these precepts your life will be prolonged." I quote this to show you the character of this faculty, and that their advice could not be much improved upon in this day.

In the thirteenth century, the king of Naples issued an edict forbidding any one to practice medicine in his kingdom who was not a graduate of the school at Palermo. The requirements of the school were a study of three years in logic and five years in medicine. He must have attained the age of 25 years. He then received a diploma as now. The surgeon was required to devote his time when a student to anatomy, and to prepare himself especially in this department. A new era had now commenced to dawn upon Europe and the world. In the thirteenth century were established most of the universities of Europe. Soon after this the discovery of printing gave a renewed impetus to learning, and the buried treasures of the Greeks and Romans were brought to light. Knowledge was everywhere sought after, everywhere cultivated, and philosophers, statesmen, orators, and physicians, assumed the high rank to which their learning entitled them.

We have noticed the primitive period—that of Æsculapius, the priestly period, that of the Æsculapiades, the Hippocratic and Philosophic period, or the Greek period, the schools of Cos, of Pergamos and Alexandria, the Arabic period, the scattering of civilization in the destruction of Greece and Rome, the dark ages, and that of light now dawning upon us—this the great era of the world, this the *magnus ab integro soeclorum nascetur ordo*. Great advancement had been made in the periods recited, both in surgery and in the practice of medicine. Trephining is described in all its minutia by Hippocrates, and for hundreds of years was practiced by the Æsculapiades. Even the superior anatomical school of Alexandria added nothing to this operation, and it was almost abandoned by the later Greek and Latin physicians, and ointments and pomades substituted. Nasal polypus was extracted and cauterised by Hippocrates, and the tongue, and the palate, and the tonsils were scarified and cauterized. One of the Æsculapiades even performed tracheotomy. Hippocrates performed the operation of empyema, and boldly punctured the chest for collections of pus or serum. The operation, however, fell into disuse afterwards, and was not practiced by the Greek, Latin, or Arabic physicians, and in fact was not revived until about the sixteenth century. He practiced paracentesis abdomino—*directs* that the puncture must be made near the navel. He did not cut for stone in the bladder, but says he abandoned that operation to the mercenaries who devoted themselves to it, which is significant that this capital operation was not only known, but practiced in the early age of medical science. This operation was also long abandoned, but was performed by the professors at Alexandria, and by Celsus at Rome, and by itinerants in the middle ages, until it came to be practiced again by the celebrated surgeon Guy de Chanlias Celsus. Galen and Leonidas, of Alexandria, treated hydrocele by excision, by the seton, cauterization, and puncture. All that our age has done is to add that of stimulating injections. The ancients were close observers of disease, and of all the causes inducing them, such as atmospheric changes, habits, manners, alimentation, and all other causes relating to health and disease, and we are astonished that prior to the study of anatomy, they practiced surgery as boldly and successfully as they did. Scarcely a disease now known but that was accurately described by Hippocrates,

Celsus, or Galen, and the zeal with which medicine was taught and practiced in the schools of Cos, of Pergamos, and Alexandria, may justly excite the wonder, and command the admiration of all ages and all men. But, upon the revival of letters, and the discovery of printing, and the establishment of medical schools throughout Europe, all that had been taught and known before was taught again, and although the improvement was slow, it was upon a sure and more substantial basis. Although they adhered to the doctrines of Hippocrates, Plato, Aristotle, Galen, Rhazes, and Avicenna, in all the schools until about the sixteenth century, still the amphitheatres for dissection and the laboratories of chemistry opened up new avenues of knowledge, which gave increased intelligence and knowledge to the medical world. Necropsy came into practice, and pathology made a beginning to give assurance to observation. Chemistry was rudely known as early as the third century as alchemy, the only object of which seemed to be change, or attempt the change of the baser metals into gold and silver, but really did not become a science until the last century, since which it has advanced more rapidly, perhaps, than any other branch of our science.

It was reserved for the sixteenth century to throw off adherence to ancient doctrines and dogmas, and to take a rapid and long step in the march of improvement. It was an age of regeneration in science, and while the torch of physical science was making the world brilliant by the discoveries of Gallileo, by his first calculated laws of gravity, by his discovery of the weight of the atmosphere, and his discovery of the movement of the earth on its own axis, and around the sun; William Harvey discovered the circulation of the blood, and Malpighi, a few years later, in 1661, demonstrated for the first time, by the aid of the microscope, the progression of blood globules in the small vessels; and Hallar and Helvetius instituted experiments to calculate and determine the forces and the manner by which the movements of the chest are affected. About the middle of the same century, lymphatic vessels and their functions were discovered by Picquet, which confirmed the doctrine of Harvey and overthrew that of the ancients, which attributed to the liver the functions of hæmatosis. Comparative anatomy was carefully studied, and the structure of the nervous system better known. About the beginning of the seventeenth century, the celebrated mathematician, Kepler, announced that the chrystaline lense was not, as had been supposed until that time, the seat of vision, but that its function is to refract the rays of light. The researches of Sir Isaac Newton on light and color contributed also to perfect the theory of the visual functions. The organ of hearing and its internal muscles and minute bones were carefully studied, and Duverney and Vicussins, by comparative anatomy, corrected former errors, and established the true seat of audition in the membrane which lines the drum and labarynth. Thomas Willis regarded the brain as an assemblage of various apparatuses, and assigned special functions to some of its divisions, and Camper, by the comparison of heads of men and animals, established the facial angle as the criterian of intellect. Showing that as we descend from the higher to the lower animals, the forehead recedes and the jaws become more elongated. The vital forces attracted attention in this age, and while the mathematicians assumed to explain the functions of the animal economy by the laws of mechanics—that the secretions, the circulation and nutrition, were the result of the elasticity of the tissues and of the friction of liquids; the chemical physician explained it upon the principle of mixture of chemical elements, of alkalies, acids, gases, salts, and fermentations. But Francis Glisson, an Oxford professor, overthrew these fallacies by the recognition of irritability as a principle or part of the living solid tissues. In 1757, Haller published his great work on Physiology, and henceforth this branch of our science has held a separate existence from physics or chemistry, and it was demonstrated that life has its laws and its special forces, which require a particular method of study. In the seventeenth century, Cheselden, the Monroes, and Bœrhave, made wonderful advances in surgery, anatomy, and physiology, and in our own time their works are standard authority. The next century, the eighteenth, presents us with the most brilliant array of names that have ever shone upon the world of medical science. Brown, Cullen, Hunters, the Gregorys, Bichat, Lind, Pringle, and Jenner. Jenner, who has made himself immortal by the introduction of the cow-pox to protect the human family from the dreadful scourge which prior to that annually swept to the grave nearly half a million of the European population, and mutilated and disfigured as many more; Howard, the prison reformer, and Count Rumford, William Thompson, of New Hampshire, the Bavarian Lieutenant-General and philosopher, who gave bread and labor to the poor by his improvements, were of this age, and co-workers in medical reform. A long list of other names might be added, which would form a galaxy more brilliant than any the world has ever seen.

A division in the various branches of medicine now came into more general use, and men devoted themselves to special departments, and consequently a higher degree of perfection obtained than had hitherto. Abercrombie, upon the Brain; Læennec, upon Diseases of the Chest; Hunter, upon Anatomy, Surgery, and (Syphilis;) Good, on Practice; Bird, on the Urinary Organs; Lawrence, Scarpa, Travers, and Richter, on Operative Surgery, and Diseases of the Eye; Cooper on Surgery; Desault and Bichat, the former on Surgery, and the latter upon the Membranes, Life and Death, and upon Anatomy and Physiology. A mere recital of these great names, familiar to us all as household words, sufficiently indicate the progress of medical science in their age. It was the era of intellectual growth and progression. It was the Augustan age of medical science; the crowning glory of all preceding epochs, and the volcanic upheaval of slumbering minds from the womb of time, as the precious metals are thrown up from the deep caverns of earth. *Salve magna parens virum.* In our own country, we have had Physick, and Rush, and Warren, to represent this age. The present century has not lost the impetus given by the last, and improvements have been made in the pathology and treatment of diseases that now impress us as perfect, but which, no doubt, in the future, and in a more advanced stage of medicine, will be found as imperfect as many of the adopted theories and practice of former times, which have yielded to advancement. Since most of those before us have entered the profession, especial improvement has been made in the treatment of diseases peculiar to women; and for this we are indebted to no one so much as to Bennett, whose researches (in uterine pathology) have excelled those of Montgomery and Kennedy, and, in fact, all others. The pathology of inflammation and congestion has induced a most wonderful change in the treatment of diseases within the last few years, and the physician can now prescribe with more assurance to himself and satisfaction to his patient. He knows what he does; he travels a plain and well-known road in the bright light of a noonday sun. He discriminates and comprehends the pathological conditions before him, and treats inflammation as such, and congestion as such. When the Humoralists and the Solidists contended for supremacy in their respective doctrines, each one attributed all morbid phenomena to his own theory. Now, full credit is yielded to the influence of each and all the parts and elements of the human body—all necessary to life; all suffering, directly or indirectly, in disease.

Our researches are conducted with more philosophy. We study by analysis and synthesis. We separate to ultimate and known parts, and again combine in order; study and view all separately, and then combine and study the whole—their action, relation, and dependence one upon the other.

Men of sense no longer attribute to a single cause all disease, and to a single medicine a cure for all. The blood has been studied to its ultimate elements. The solids have been studied in like manner; and the action of remedies upon each have been thoroughly tested. But from these various *isms* light has been shed upon the science. From Homœopathy the profession has learned to give less medicine, and with greater exactness. From Hydropathy we have learned the value of water, externally and internally. But a few years since, the fevered patient thirsted, but was excluded from drink. In fact, in all ages, God's freest and best gifts, air and water, have been excluded from use to an extent unwarranted by science or common sense. From the

Thompsonian doctrine we have learned something of the value of the application of heat and moisture and stimulating applications. Hence, theories and doctrines which seem foolish and and unwise have their agencies to perform in working out good. Even to combat them requires thought and investigation. We have ourselves seen a change in pathology and therapeutical applications in almost every disease. We have witnessed the struggle and supremacy of science over ignorance; all of which has given a large per cent. of the saving of life over former years. The pathology and treatment of every variety of fever, of pneumonia, of dysentery, and, in fact, of nearly every disease, has changed, or undergone great improvement. WATSON, and TODD, and GRAVES, and BENNETT, and PARKER, RICORD, STANLEY, and WILLIAMS, and a thousand other glorious names, have given a new era in their respective fields of study and investigation.

Genius and learning is concentrated to special departments of our science; that has given a perfection that could not have been expected from universal devotion to the expanded fields, too large for the comprehension of any one mind; too extended for the brief span of life allotted to man.

And now, gentlemen, what is the duty devolving upon us? Clearly, it is to commence the great unfinished work of those who have preceded us, and to carry it on to the glory and honor of our profession, and to the welfare and happiness of mankind. Each one should be *vir probus medendi peritus*—an honest man instructed in the art of healing. He should be an earnest man with enlarged and catholic views in all that pertains to learning; in all that pertains to human life, and human disease. He should remember that his own sphere, whether in the country, the hamlet, or village, requires the same knowledge, the same thought, as that of those in the great cities of the earth: for the same complex diseases and accidents are presented for his treatment, his investigation, his decision and management, to those. Locality does not change the size of the brain, does not affect thought, but may stimulate it to action, and excite it to labor. All may enjoy alike the experience and the ideas of all preceding ages, and by reflection and the cultivation of the judgement, may analyze, arrange, and use them to advantage.

*Practical medicine* involves the knowledge of all that is known of the science of medicine, and whether good or bad, depends upon the proper use of that knowledge.

We have yet much to learn, and we should, and may, all become investigators, improvers, and teachers. Specific inflammation will in all probability be extended beyond its present sphere. That of rheumatism, of syphilis, and variola, may be made applicable to other forms now regarded simple, but differing from them. Hygiene will be improved, an increased knowledge of atmospheric influences will aid us in bringing to light causes of disease now unknown. The now hidden mysteries of epidemics will yet be made plain, simple, and appreciable.

The bloody war from which we have just emerged has given no less luster to hygiene, alimentation, and the treatment of wounds induced by projectiles of improved character, than it has to the settlement of constitutional liberty. This age is one of progression; the fire of knowledge is consuming the accumulated rubbish of ages, and while the heavens and the earth are lit up by it, let us individually, and as a society, seize upon it, and make it brighter and brighter, until disease shall acknowledge our strength and power, and man shall go to the end of his three-score years and ten without its impairment, and shall die alone from expended vital force, and the fiat of the God who made and controls and governs all things.

---

Havard Medical College.—Dr. James C. White, the talented junior editor of the *Boston Medical & Surgical Journal*, has retired, in order to accept an adjunct professorship of Medical Chemistry, in the Harvard Medical School.

Milk Consumed in New York and Vicinity.—The milk consumed in the city of New York and its immediate vicinity, is estimated at an average of three thousands quarts a day.

Death of Dr. W. Brinton.—Dr. William Brinton, F.R.S., the distinguished London physician, died of uræmia, on the 18th of January, aged 43 years.

